# Origins, timing and introgression of domestic geese revealed by whole genome data

**DOI:** 10.1186/s40104-022-00826-9

**Published:** 2023-02-13

**Authors:** Junhui Wen, Haiying Li, Huie Wang, Jincheng Yu, Tao Zhu, Jinxin Zhang, Xinghua Li, Zhihua Jiang, Zhonghua Ning, Lujiang Qu

**Affiliations:** 1 Department of Animal Genetics and Breeding, National Engineering Laboratory for Animal Breeding, College of Animal Science and Technology, China Agricultural University, Beijing, China; 2 College of Animal Science, Xinjiang Agricultural University, Urumqi, China; 3 Xinjiang Production & Construction Corps Key Laboratory of Protection and Utilization of Biological Resources in Tarim Basin, Alar, Xinjiang, China; 4 College of Animal Science, Tarim University, Alar, Xinjiang, China; 5 Liaoning Academy of Agricultural Sciences, Shenyang, China; 6 Department of Animal Sciences, Washington State University, Pullman, USA

**Keywords:** Domestication, Goose, Introgression, Phylogeny

## Abstract

**Background:**

Geese are among the most important poultry species in the world. The current generally accepted hypothesis is that the European domestic geese originated from greylag geese (*Anser anser*), and Chinese domestic geese have two origins, most of which originated from swan geese (*Anser cygnoides*), and the Yili goose originated from greylag geese. To explain the origin and demographic history of geese, we selected 14 goose breeds from Europe and China and wild populations of swan and greylag geese, and whole genome sequencing data were obtained for 74 samples.

**Results:**

Population structure analysis and phylogenetic trees showed that the wild ancestor of Chinese domestic geese, except for Yili, is the swan geese, and the wild ancestor of Chinese Yili and European domestic geese is greylag geese. Analysis of the demographic history suggests that the domestication of Chinese geese occurred ~ 3499 years ago and that of the European geese occurred ~ 7552 years ago. Furthermore, gene flow was observed between domestic geese and their wild ancestors. Analysis of introgression showed that Yili geese had been introgressed by Chinese domestic geese, and the body size of Yili geese may be influenced by introgression events of some growth-related genes, including *IGF-1*.

**Conclusions:**

Our study provides evidence for the origin of geese at the genome-wide level and advances the understanding of the history of goose domestication and the traits affected by introgression events.

**Supplementary Information:**

The online version contains supplementary material available at 10.1186/s40104-022-00826-9.

## Background

Animal domestication has led to the development of urban communities and expansion of agricultural economies. It is an important event in human history and has contributed to the progression of human civilizations [1]. Domestication is the process by which humans select animals that meet their needs and preferences, causing domesticated animals to differ from their wild ancestors [2]. Domestication syndrome is a common characteristic of domestic animals [3], and includes phenotypical, behavioral, and production variations. These variations have been selected to correlate with human needs, such as meat, eggs, and milk as major sources of high-quality protein. The genetic mechanisms underlying variation due to domestication have been investigated using molecular techniques in a large variety of domesticated animals, including dogs [4], goats [5], cattle [6], chickens [7], and ducks [8].

Domestic geese (family: Anatidae, order: Anseriformes) are economically important poultry [9]. Romanov proposed six centers of domestication, breed formation, and dispersal for domestic geese based on genetic, phenotypic, phylogenetic, and historical analyses: Western Europe, China, Eurasia, Egypt, North America, and Australia [10]. Geese were recorded in ancient Egyptian paintings (2686–1991 BC) [11]. Ancient Greek and Roman written records include mentions of domestic geese. Romans consumed goose meat and eggs, produced fatty liver, and used goose feathers to make goose quills and ornaments [12]. The domestication of geese in China can be traced back to the Neolithic period (5000 BC), as evidenced by a goose-shaped artifact from that period [13]. In 2022, goose bone fossils were found in a 7000-year-old rice-cultivation village in the lower Yangtze River, China [14]. After a long period of domestication, China has had abundant domestic-goose breeding resources. In 2020, China published a national list of livestock genetic resources, including 30 Chinese goose breeds [15].

**Fig. 1:**
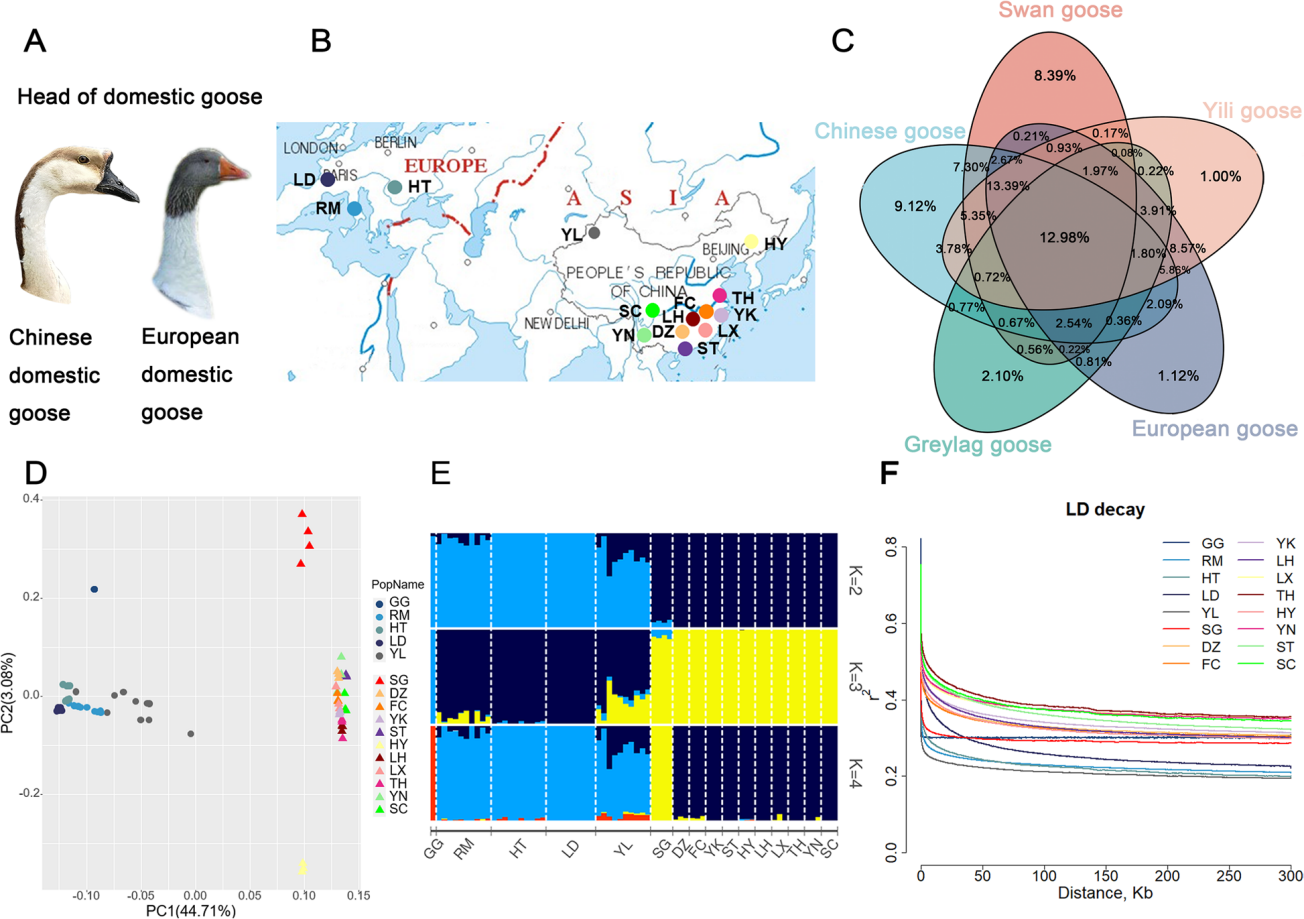
Experimental setup and population genetic structure. **A** Knob on head of domestic geese, the Chinese domestic goose is on the left, and the European domestic goose is on the right. **B** Location of the samples used for this study. A total of 74 geese, including swan geese (*n =* 4; origin, China), greylag goose (*n =* 1; origin, Europe), 11 Chinese goose breeds (*n* = 10 of YL; *n =* 3, each a different breed), and three European domestic goose breeds (*n =* 29) were used. See Additional file 1: Table S1. **C** Genomic SNPs of five populations. **D** PCA plot of geese breeds. **E** Population genetic structure of geese. The length of each colored segment represents the proportion of the individual genome from the ancestral populations (*K* = 2–4), population names are at the bottom. **F** LD decay patterns

The taxonomy and phylogeny of geese have been studied for more than 300 years, and traditional classification is based on behavioral traits, morphological characteristics, and anatomical structure [16]. There are clear morphological differences between the two regions of Europe and China for domestic geese. The Chinese domestic goose is relatively small and light, with high egg production [17]. It has strong legs and wings, and can forage over large distances. This may explain the extended distribution of Chinese geese beyond Chinese borders into India and Siberia, which increases the possibility of gene flow events [18]. The Chinese domestic goose has a knob at the base of its beak, while European domestic geese and Yili geese in northwest China do not (Fig. 1A). The European domestic goose is relatively large and heavy, with less egg production than the Chinese geese [18, 19].

It is generally accepted that domestic geese have two origins [9]. The ancestors of domestic geese in Europe are the greylag geese (*Anser anser*). The ancestors of domestic geese in China have two sources. Yili geese are domesticated from greylag geese, whereas all the other local Chinese geese are derived from swan geese (*Anser cygnoides*) [20]. Darwin suggested that the European domestic goose was derived from the greylag, possibly based on plumage patterns [2]. In 1968, Bhatnagar found that the karyotypes of European domestic and European greylag geese were identical, and the karyotypes of Chinese domestic and Asian swan geese were identical [21]. In 1998, Shi et al. performed restriction fragment length polymorphism analyses on mitochondrial DNA from Chinese geese and found that Yili have a different origin than other Chinese geese [22]. In 2011, Li et al. performed shared haplotype and systematic evolution analyses using the 521-bp control region (D-loop) of mitochondrial DNA from 26 Chinese domestic goose and six Landaise geese breeds and found two maternal origins of Chinese domestic geese [23]. Gao et al. performed a phylogenetic analysis using single-copy genes to study the evolution of geese in 2016, and found that wild and domestic geese clustered into a subclade, which is consistent with the hypothesis that domestic geese were domesticated from wild geese [24].

In 2020, Heikkinen et al. showed that domestication and interspecific hybridization of European domestic geese using genome-wide markers [25]. Interspecific genetic introgression, which alters traits, is also a topic of interest in domestication studies. A typical finding of introgression is the historical introgression from wild relatives enhanced climatic adaptation and resistance to pneumonia in sheep [26]. Chen et al. found historical introgression events in East Asian domestic cattle that helped them adapt to high-altitude environments [27]. There are no studies on the mechanisms of introgression and alteration of traits in domestic geese of different origins.

To elucidate the evolutionary history and introgression events of the domestic goose, we analyzed the genomes of 74 geese, including the swan goose, greylag goose, and 14 domestic goose breeds. We identified the origin of Chinese and European domestic geese at the genomic level, and inferred the timing of domestic goose domestication. Additionally, our study identified substantial evidence of gene flow from swan goose originating from Chinese geese to greylag goose originating from Yili geese, and found that introgression could lead to changes in the body size of Yili geese.

## Methods

### Sampling

Whole blood samples were obtained from 74 geese, including 11 Chinese domestic goose breeds, three European domestic goose breeds, and their wild ancestors, the swan goose (*A. cygnoides*) (SG, *n =* 4), and the greylag goose (*A. anser*) (GG, *n =* 1), whose data were acquired from a previous study [28]. Domestic geese have two origins. Ten Chinese domestic goose breeds are thought to have originated from the swan goose, including Daozhou grey goose (DZ, *n =* 3), Fengcheng grey goose (FC, *n =* 3), Yongkang grey goose (YK, *n =* 3), lion-head goose (ST, *n =* 3), Huoyan goose (HY, *n =* 3), Lianhua white goose (LH, *n =* 3), Linxian white goose (LX, *n =* 3), Taihu goose (TH, *n =* 3), Yunnan goose (YN, *n =* 3), and Sichuan white goose (SC, *n =* 3). A Chinese domestic goose breed, Yili goose (YL, *n =* 10), from Northwest China, Xinjiang, and three European domestic goose breeds, Roman geese (RM, *n =* 10), Hortobagy goose (HT, *n =* 10), and Landaise goose (LD, *n =* 9), are regarded as originating from the greylag (Additional file 1: Table S1). The data of Roman and Hortobagy geese were downloaded from the National Center for Biotechnology Information (NCBI, BioProject ID: PRJNA722049).

### Sequencing and variant calling

Genomic DNA was extracted using the standard phenol/chloroform extraction method [29]. The quality and integrity of the extracted DNA were verified using a NanoDrop spectrophotometer (Thermo Fisher Scientific, Wilmington, DE, USA). Paired-end (150 bp) libraries were sequenced and built using the Illumina Novaseq 6000 platform, according to the manufacturer’s instructions. Quality control of raw sequencing data was performed using the NGS QC Toolkit (v2.3.3) with the default parameters [30].

Paired-end reads were mapped to the goose (*Anser cygnoides domesticus*) genome version 1.0, using BWA (v 0.7.17) [31, 32] with default parameters. The alignments were sorted and duplicate sequences were removed using Picard [33]. The reads were realigned using RealignerTargetCreator and IndelRealigner in GATK (v3.8) [34] to reduce the error rate of alignment. Variant calling was performed using UnifiedGenotyper in GATK (v3.6) with a minimum base quality of 20. Variant filtering was performed using VariantFiltration in GATK (v3.6) with the recommended parameters. After filtering, 24,891,622 SNPs and 2,885,072 indels were obtained and used in the subsequent analyses.

### Population structure

VCFtools software (v0.1.16) [35] and PLINK (v1.90) [36] was used to convert the VCF file to the PLINK format. Stringent filtering criteria were applied with the following parameters: --geno 0.1, --maf 0.1, and --indep-pairwise 20 10 0.5. After filtering, 11,410,883 variants were obtained for principal component analysis (PCA) with PLINK (v1.90). This analysis was performed using default parameters to extract the top 20 principal components of the variance-standardized relationship matrix.

The filtered dataset of 11,410,883 variants was used to analyze the admixture of each individual and population clustering using ADMIXTURE (v1.3.0) [37]. The VCF file was converted to the ADMIXTURE input file format using PLINK (v1.90). The analysis was performed by setting the assumed number of ancestral populations, *K*, from 2 to 16. Cross-validation (CV) was performed with a five-fold value to verify the optimal ancestral populations. When the CV error value was the lowest, the corresponding *K* value represented the optimal number of ancestral populations. The final population clustering and individual admixture results were visualized using Pophelper package [38].

Phylogenetic tree reconstruction was performed according to the SNPhylo protocol [39]. The VCF files were converted to HapMap format using custom Perl (v5.32.0) scripts. Maximum likelihood (ML) trees were constructed using DNAML programs in PHYLIP (v3.697), with bootstrapping analysis performed with Phangorn. The phylogenetic trees were visualized using iTOL [40].

PopLDdecay software was used to calculate the linkage disequilibrium (LD) decay of the 16 geese breeds [41]. The SNPs were filtered using the following parameters: -MAF 0.05 and -Miss 0.25. Then, the LD measurement *r* ^2^ was calculated using the filtered dataset and a final LD decay plot was generated.

### Demographic analysis

Population demographic history analysis of domestic geese and their wild ancestors was performed using the SMC++ [42]. The parameters for this analysis were set based on the goose genome and studies from other birds to an average mutation rate (*μ*) per generation of 1 × 10^−9^ and generation time (*g*) of 2 years [43].

Demographic inferences were made using DaDi [44]. To avoid the effect of selection on the demographic analysis, we focused on non-coding regions and extracted non-coding SNPs. The final dataset contained 23,080,074 SNPs, spanning 1,041,100,496 bp. To avoid the linkage of SNPs, we thinned the non-coding SNPs to 1% and obtained a dataset of 230,801 SNPs. Owing to the unknown ancestral state of each SNP, we folded the frequency spectrum. The projection value was selected based on the strategy of maximizing the number of segregated sites.

We tested several models to estimate the demographic history of Chinese and European domestic geese and the gene flow which occurred during domestication. As the Yili and European domestic geese share the same origin (see results), we added the Yili goose samples to the European domestic geese group. For each model, multiple runs were performed to ensure that the parameters converged to a similar log-likelihood. The starting parameter values for the first round were randomly assigned, and the best parameters obtained after completion of a round were used as the starting parameters for the subsequent rounds. After the convergence of the parameters, we retained the model with the highest log-likelihood as the final simulation result. The parameters of the optimal model were converted into absolute units using the average mutation rate per generation and generation interval. Confidence intervals for the parameters were generated using the Godambe information matrix (GIM) with 100 bootstraps [45].

### Introgression analysis

Gene migration events between populations were inferred using TreeMix [46]. TreeMix uses genome-wide allele frequency data to infer patterns of differentiation and mixing across multiple populations. The software inputs data as allele frequencies for multiple populations to infer the population migration events. The VCF file was converted to the plink format using VCFtools (v 0.1.16) and PLINK (v1.90) was used to calculate the allele frequencies. A ML tree was constructed using TreeMix, and the parameters were set at the migration events (*m* = 1).

The ABBA-BABA test, also known as the *D*-statistic, was used to infer the existence of gene flow between the populations. This analysis of *D*-statistic values was performed using Dsuite software [47], which can calculate *D*-statistics at the genome scale across all combinations of populations with VCF input files. According to the principle of ABBA-BABA test calculation, (P1, P2, P3, O) represent four different groups: P1 is a domestic goose group, P2 is another domestic goose group and sister group with P1, P3 is a closely related species of P1 and P2, and O is the outgroup. Gene flow between domestic geese and their wild ancestors was inferred with the mallard as an outgroup. Dtrios was used to calculate the *D* and *f*_4_-ratio statistics for all trios of populations in the dataset, with a default value of 20,000 for the Jackknife block size. Then, using the Dinvestigate program to calculate the *D*-value for windows containing useable-size SNPs, the sliding window consisted of 2500 SNPs and a step of 500 SNPs. The locations of the windows with the top 5% *D* values were obtained and the genes in these windows were regarded as candidate genes for introgression. Functional annotation of the obtained gene dataset was performed using DAVID (v6.8) [48]. Gene ontology (GO) categories were visualized using R (v3.6) [49].

The degree of introgression in Yili goose individuals and the direction of introgression between Yili and other Chinese domestic geese were detected by calculating the *F*_ST_ between Yili individuals and European domestic geese and the *F*_ST_ between Yili individuals and other Chinese domestic geese, respectively, with a sliding window of 100 kb and a step of 50 kb. The ML tree was constructed for the candidate fragments using MEGAX software [50].

The potential mechanisms affecting body size in geese influenced by introgression were evaluated using the gene and 5′ regulatory region sequences of *IGF-1* (insulin-like growth factor-1) in geese from NCBI and a study by Wang et al. [51]. The 5′ *IGF-1* regulatory region sequence was obtained from the reference genome using BLAST [52], the 5′ *IGF-1* regulatory-region SNP dataset of 74 samples were extracted, and JASPAR was used to predict the transcription factor-binding sites [53].

## Results

### Sequencing and variations

Whole-genome resequencing of the swan geese, the greylag geese, and 14 domestic goose breeds of two origins (Fig. 1B) was performed, and sequences were mapped to the swan goose genome (v1.0). After quality control and filtering, 923.6 Gb of high-quality sequences were obtained, with an average of 12.5 Gb per individual. Across all samples, a total of 5938 million mapped reads was obtained, with an average depth of 11.2X and an average coverage of 95.8% per individual. After variant calling, a total of 27.78 million variants was obtained, including 24.89 million SNPs and 2.885 million indels. The genomic SNPs of the five populations (swan geese, Chinese domestic geese, greylag goose, European domestic geese, Yili geese) are shown in Fig. 1C. Yili geese had 72% of its SNPs in common with that of the Chinese domestic geese and 81% in common with that of the European domestic geese.

### Population structure

The PCA indicated clustering between domestic geese and their wild ancestors (Fig. 1D). In the first principal component, most Chinese domestic geese (DZ, FC, YK, ST, HY, LH, LX, TH, YN, and SC) and swan geese clustered, while Yili geese, European domestic geese (RM, HT, and LD), and greylag goose clustered. The PCA also showed divergence within the clustering; the Yili geese deviated towards other Chinese domestic geese in the first principal component and the wild ancestors separated from domestic geese in the second principal component.

Further, the population structure was inferred using ADMIXTURE to deduce the population admixture proportions by assigning ancestral populations *K* from 2 to 16*.* At *K* = 2 (CV error = 0.47774), we observed two separate clusters: Chinese domestic geese (DZ, FC, YK, ST, HY, LH, LX, TH, YN, and SC) and the swan geese formed one cluster, while Yili, European domestic geese (RM, HT, and LD), and the greylag goose formed the second cluster. At *K* = 3 (CV error = 0.47738), CV error was the lowest, three clusters were observed, the greylag goose clustered alone, European domestic and Yili geese clustered, and the swan geese and other Chinese domestic geese (DZ, FC, YK, ST, HY, LH, LX, TH, YN, and SC) clustered. At *K* = 4 (CV error = 0.50603), four clusters were observed: greylag goose, swan geese, and Chinese domestic geese (DZ, FC, YK, ST, HY, LH, LX, TH, YN, SC) clustered separately, European domestic geese and Yili clustered while showing different degrees of mixing from the greylag goose and Chinese domestic geese (Fig. 1E).

Genome-wide LD statistics were plotted for the 16 populations (Fig. 1F). The LD decay rate of domestic breeds subjected to long-term selection was slower than that of their wild ancestors, the swan geese and greylag goose showed the fastest LD decay and the smallest *r* ^2^, indicating that they had higher diversity as wild ancestors.

**Fig. 2:**
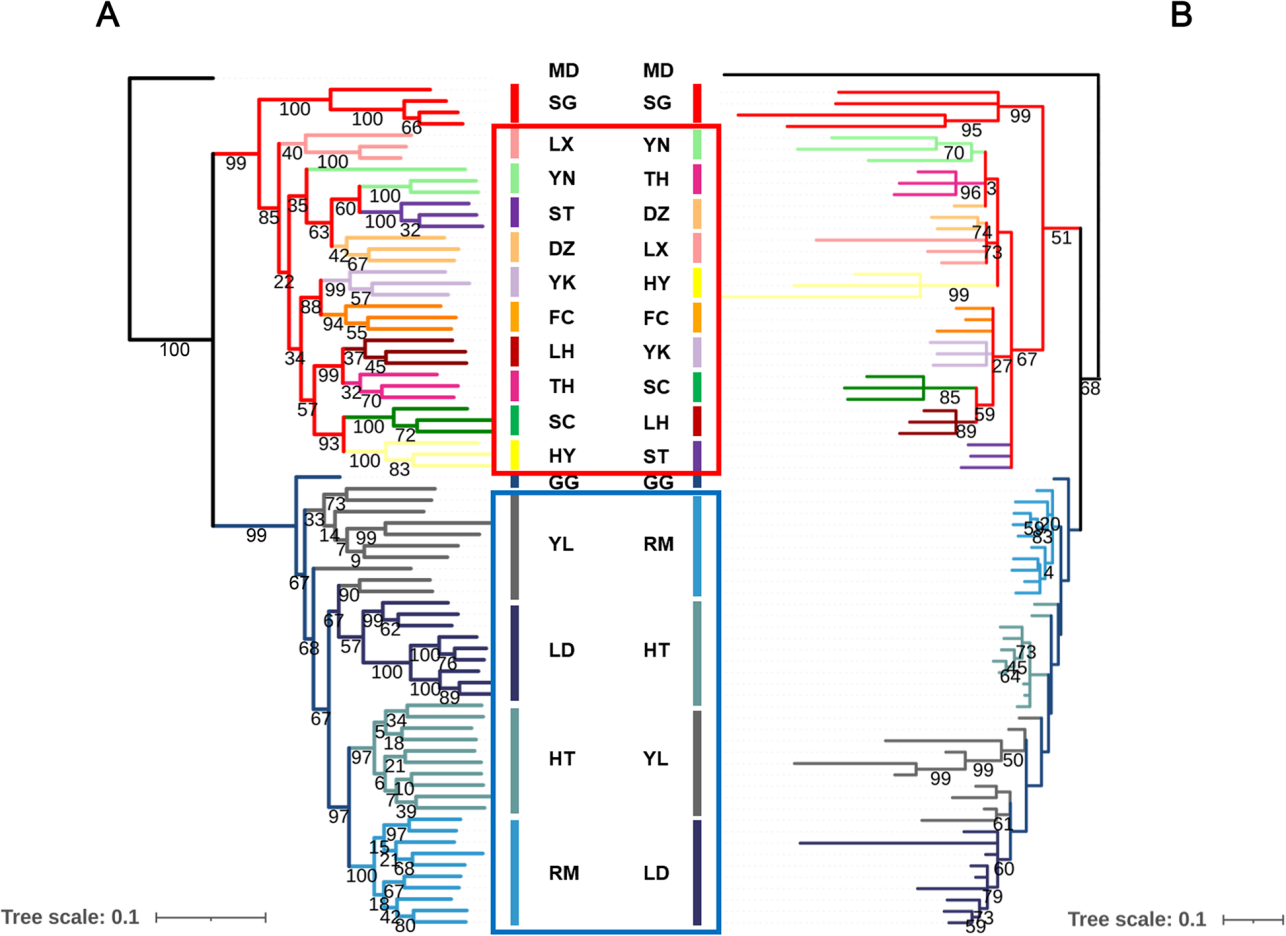
Maximum likelihood phylogenetic tree of the goose population, showing the two origins of domesticated geese. **A** Genome-wide phylogenetic tree. **B** Mitochondrial phylogenetic tree. The color of the branches indicated the different breeds of geese. The value on the branches indicated the bootstrap values. MD represents the mallard (*Anas platyrhynchos*), the outgroup used in the phylogenetic analysis. SG represents the swan geese, GG represents the greylag geese, the blue box indicates European domestic and Yili geese, and red box indicates Chinese domestic geese

The phylogenetic relationships between individual samples were inferred based on the ML trees constructed, with 1 ML tree constructed using whole genome SNPs and the other using mitochondrial SNPs (Fig. 2). In the genome-wide tree, Yili, European domestic geese, and greylag goose clustered, and the Chinese domestic geese and the swan geese clustered. The clustering observed in the mitochondrial tree supports the above results.

In conclusion, we obtained genomic evidence that most Chinese domestic goose breeds originated from the swan goose, while Yili and European domestic geese originated from the greylag goose. The Yili geese was mixed with other Chinese domestic geese.

### Demographic analysis

The SMC++ method was used to estimate changes in the historical effective population size (N_e_) of the goose populations (Additional file 2: Fig. S1). The effective population size of both wild and domestic geese showed contraction and expansion with time, and domestic geese of two origins showed concordant demographic trajectories with their wild ancestors, respectively.

**Fig. 3:**
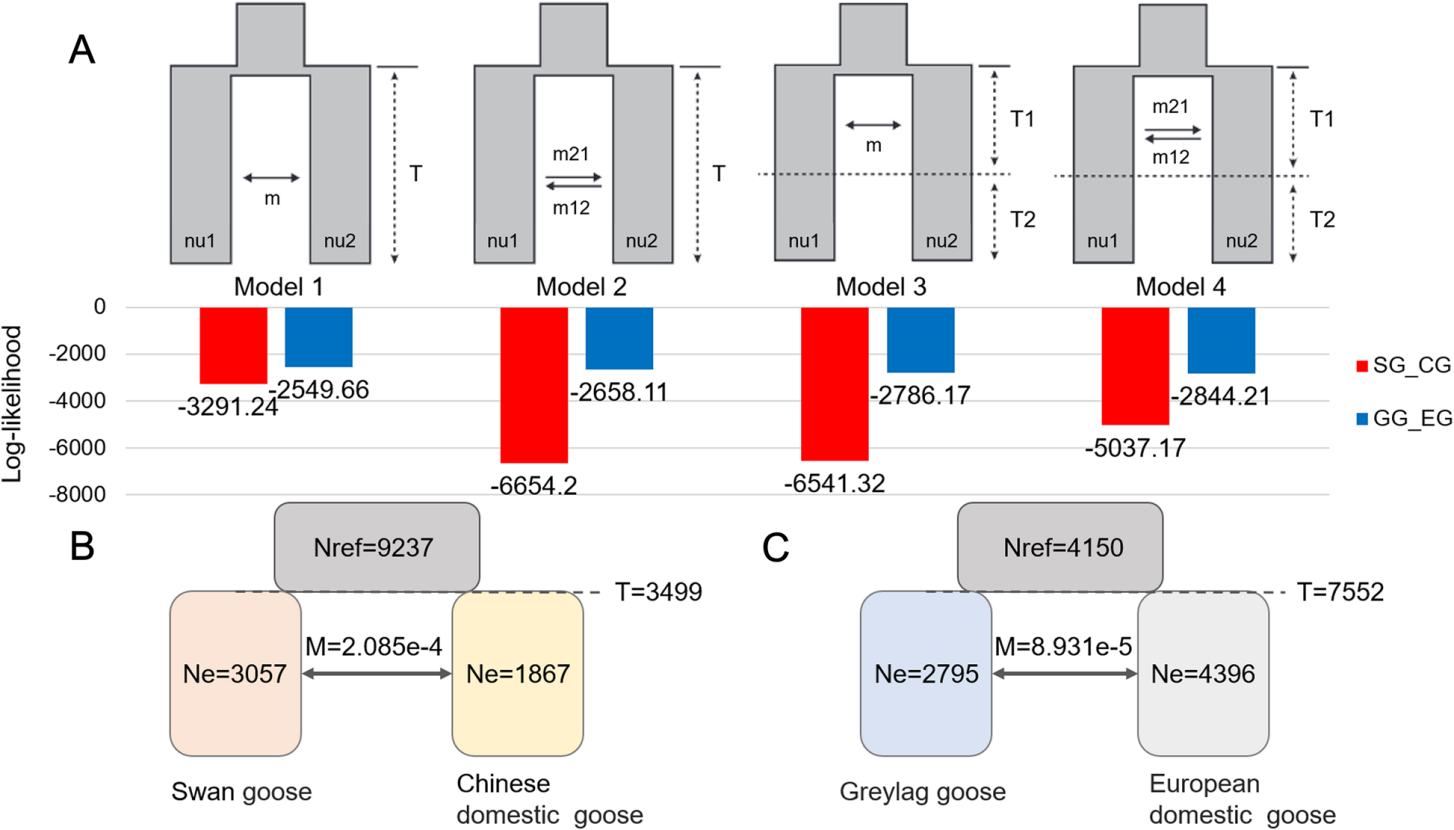
Estimation of demographic parameters to show the time of goose domestication. **A** Four different demographic models and the corresponding log-likelihood values. SG_CG indicates the demographic inference of the swan geese and the Chinese domestic geese. GG_EG indicates the demographic inference of the greylag goose and European domestic geese. **B** Best-fit estimates of parameters for the Chinese domestic goose domestication model. **C** Best-fit estimates of parameters for the European domestic goose domestication model. The time units are years and migration event units are number of migrants per generation

The demographic history of Chinese and European domestic geese was inferred by selecting four models (models 1–4). Model 1: split into two populations with symmetric migration; model 2: split into two populations with different migration; model 3: split with symmetric migration followed by isolation; and model 4: split with asymmetric migration followed by isolation (Fig. 3A).

For parameter estimation of the demographic history of Chinese domestic geese (Fig. 3B, Additional file 2: Fig. S2), model 1 had the highest log-likelihood and the lowest Akaike Information Criteria (AIC) value, indicating the best fit of the model to the data (log-likelihood = −3291.24; AIC = 6590.48). The demographic parameters estimated using model 1 indicated that Chinese domestic geese and swan geese diverged 3499 years ago with a 95% confidence interval (CI) of ±120 years (95% CI ± 120). The N_e_ values of swan geese and Chinese domestic geese were 3057.302 (95% CI ± 51.399) and 1866.709 (95% CI ± 46.379), respectively. Gene flow estimation between swan geese and Chinese domestic geese revealed 2.085 × 10^−4^ migrations per generation (95% CI ± 2.152 × 10^−7^).

For parameter estimation of the demographic history of European domestic geese (Fig. 3C, Additional file 2: Fig. S3), model 1 was the best fit to the data (log-likelihood = −2549.66; AIC = 5107.32). Model 1 indicated that symmetric gene flow existed between European domestic geese and greylag goose after domestication. Demographic parameters estimated that European domestic geese and greylag goose diverged 7552 years ago (95% CI ± 10.574). The N_e_ values of greylag goose and European domestic geese were 2795.154 (95% CI ± 11.206) and 4395.884 (95% CI ± 4.696), respectively. Gene flow estimation showed 8.9309 × 10^− 5^ migrations per generation between greylag goose and European domestic geese (95% CI ± 4.295 × 10^−7^).

### Introgression analysis

**Fig. 4:**
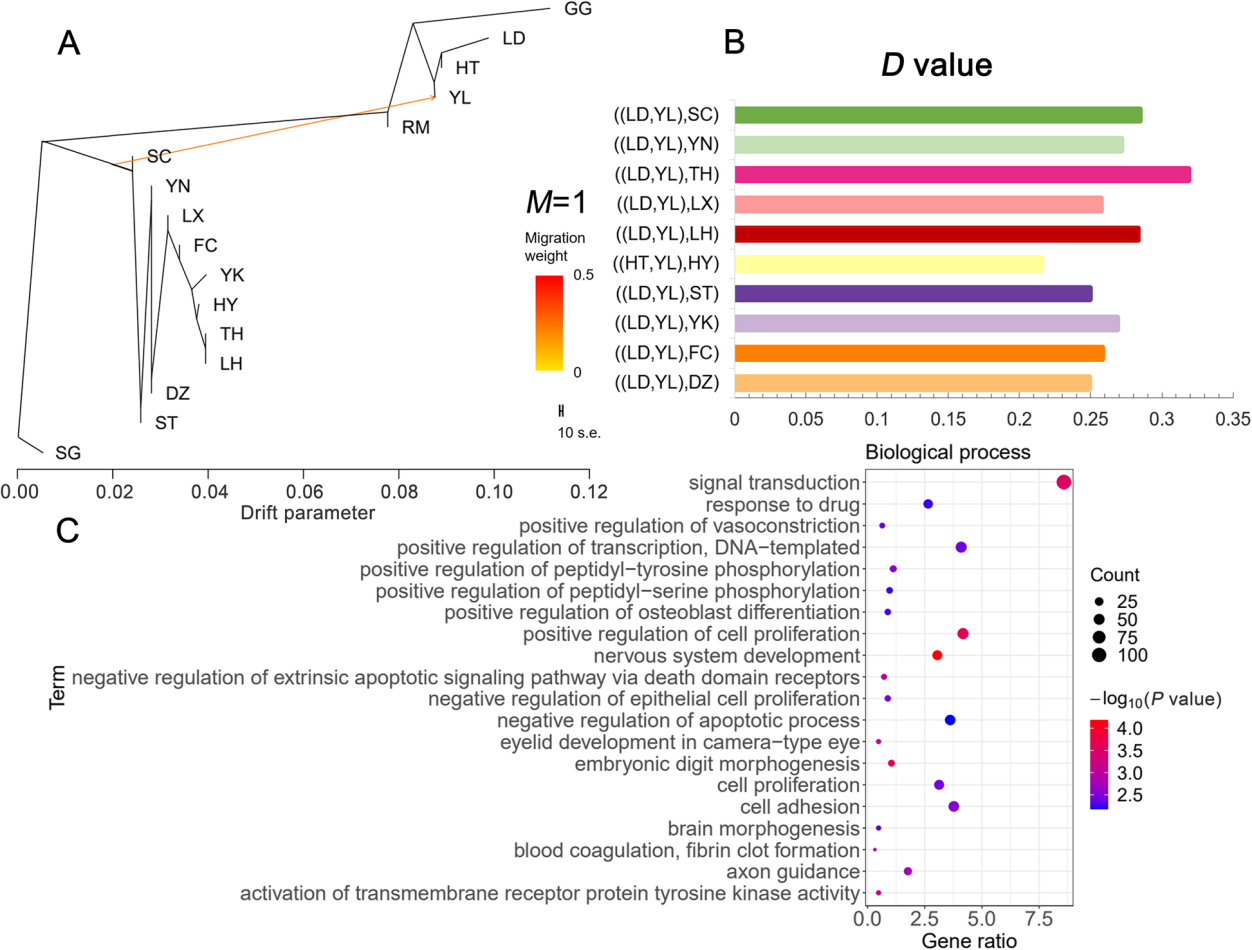
Introgression analysis of goose population. **A** Genetic migration inferred using TreeMix (*M* = 1). **B** *D*-statistics for introgression between Yili goose breed and Chinese domestic goose populations. More details in Additional file 1: Table S4. **C** GO enrichment analysis of candidate genes. The plot is shown with the top 20 significant biological processes

Migration events between populations were estimated using TreeMix and constructing ML phylogenetic trees. The results showed that gene flow from Chinese domestic geese to Yili geese occurred when the migration event was set to 1 (*M* = 1). This result corroborated the PCA and ADMIXTURE results that demonstrated the existence of admixture between Yili and other Chinese domestic geese (Fig. 4A).

To further analyze introgression events, we performed *D* statistics for all individuals based on SNP frequency differences. To detect introgression of Chinese domestic geese to Yili geese, we set (P1, P2, P3, O) as (European domestic geese, YL, Chinese domestic geese, mallard) (Fig. 4B, Additional file 1: Table S4). The *D* statistics showed significant introgression events (Z > 3, *P* < 0.001) from all 10 Chinese domestic goose breeds to Yili geese, with (LD, YL, TH) having the largest *D* value of 0.318905, indicating the strongest introgression from the Taihu geese to Yili geese. This was followed by (LD, YL, SC) with a *D* value of 0.284785, that shows, introgression from Sichuan geese to Yili geese.

The introgression region was further defined by identifying 1265 genes in the top 5% *D*-value window positions and presuming these to be candidates for introgression (Additional file 1: Table S5). GO analysis was performed on candidate genes, resulting in 227 GO enrichment terms (Additional file 1: Table S6), and the top 20 significant GO entries are shown in Fig. 4C. GO analysis revealed that genes involved in introgression were enriched in 191 biological processes, including positive regulation of cell proliferation, positive regulation of osteoblast differentiation, skeletal system development, muscle organ development, and hindlimb morphogenesis. These results suggest that the change in body size of Yili geese may be attributed to the introgression of Chinese domestic geese into Yili geese. Moreover, we identified genes associated with body size alterations, including *IGF-1, SIRT1* (sirtuin 1)*, SALL3* (spalt like transcription factor 3)*, GJA5* (gap junction protein alpha 5)*,* and *SOX4* (SRY-Box transcription factor 4).

**Fig. 5:**
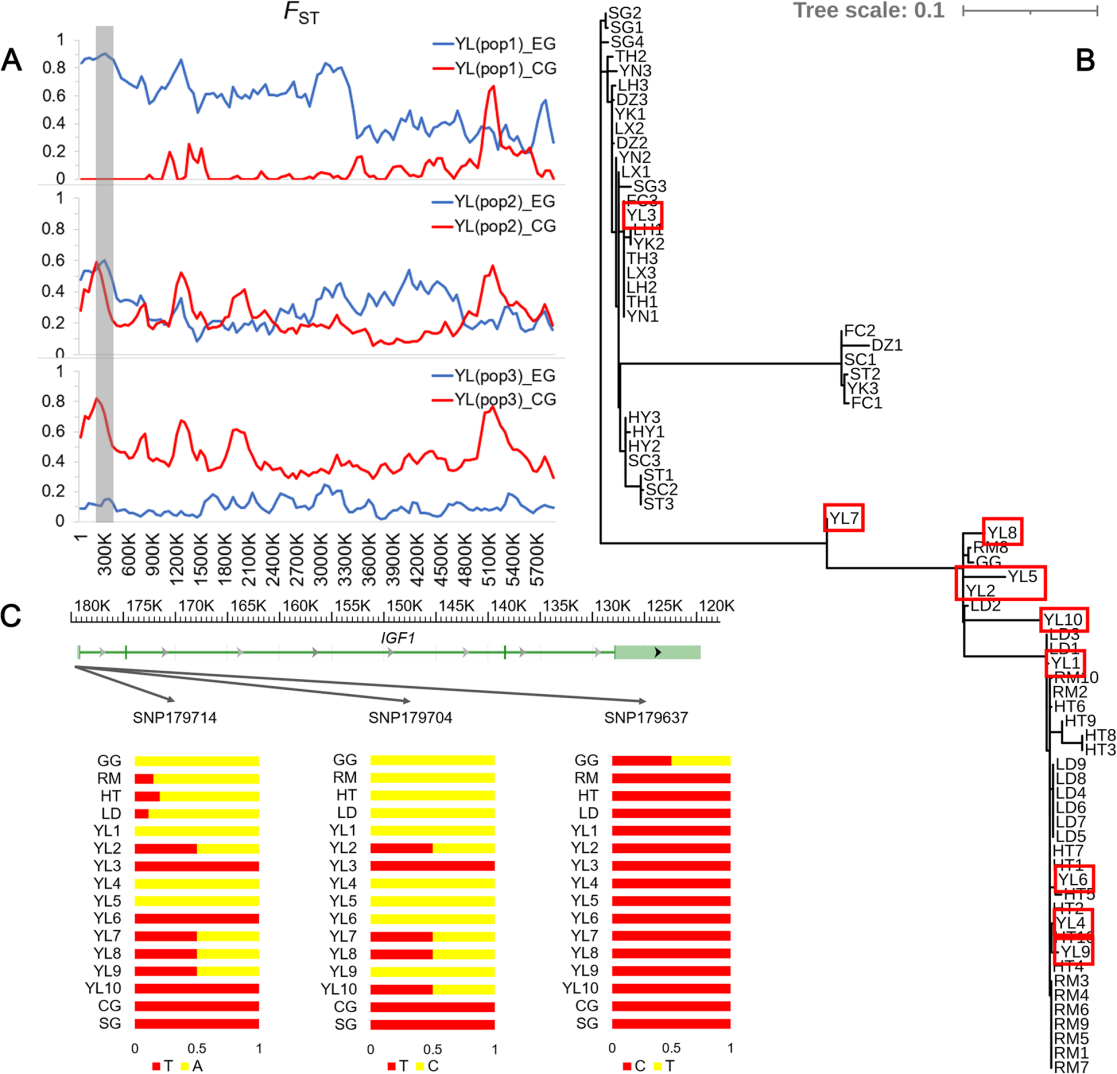
Regions with introgression between Chinese domestic geese and Yili geese individuals. **A** *F*_ST_ between Yili goose individuals and the Chinese and European domestic goose populations across scoffold NW_013185696.1. The YL goose samples were divided into three groups based on *F*_ST_ values, YL (pop1) includes YL3; YL (pop2) includes YL2, YL5, YL7, and YL8; YL (pop3) includes YL1, YL4, YL6, YL9, and YL10. The blue line represents *F*_ST_ between Yili and European domestic geese, and red line represents *F*_ST_ between Yili and Chinese domestic geese. The gray region indicates the location of the *IGF-1* gene. **B** ML tree constructed using *IGF-1* sequences of scaffold region NW_013185696.1 (119,577–179,398 bp). The red frame highlights the branches of Yili goose individuals. **C** Allele frequencies of three SNPs in the 5′ regulatory region of the *IGF-1* gene in goose populations, the SNP names represent their positions

The degree and direction of introgression in the Yili goose population was further detected by calculating the *F*_ST_ between Yili individuals and Chinese domestic and European domestic geese, respectively, based on the results of the *D*-statistic analysis (Fig. 5A). The Yili goose samples were divided into three groups based on *F*_ST_ values: YL pop1 includes YL3; YL pop2 includes YL2, YL5, YL7, and YL8; and YL pop3 includes YL1, YL4, YL6, YL9, and YL10. We obtained a region located on scaffold NW_013185696.1 (100–350 kb), which contained the candidate gene *IGF-1*. In the region, the Chinese domestic geese and YL (pop1) were poorly differentiated (*F*_ST_ = 0), European domestic geese and YL (pop1) were highly differentiated (*F*_ST_ = 0.89), YL (pop2) was moderately differentiated from both Chinese and European domestic geese, Chinese domestic geese and YL (pop3) were highly differentiated (*F*_ST_ = 0.78), and European domestic geese and YL (pop3) were poorly differentiated (*F*_ST_ = 0.10).

An ML tree was constructed using *IGF-1* gene sequences (Fig. 5B). In the ML tree, YL3 clustered with Chinese domestic geese; YL1, YL4, YL6, YL9, and YL10 clustered with European domestic geese; and YL2, YL5, YL7, and YL8 formed separate branches clustered with greylag goose and European domestic geese. Additionally, *F*_ST_ was calculated and phylogenetic trees constructed for regions where other candidate genes related to body size development were located, including *SIRT1*, *SALL3*, *GJA5*, and *SOX4*. The results of the analysis were consistent with those above, which also showed differences in Yili geese individuals (Additional file 2: Fig. S4–6). It is further suggested that there were introgression events in Yili geese from Chinese domestic geese, and that individual Yili geese were subjected to different degrees of introgression.

To explain the mechanism underlying the influence of introgression on body size, we studied variations in the *IGF-1* gene. The variation dataset of the coding sequence (CDS) region and *IGF-1* 5′ regulatory region was extracted; no mutations in the CDS region and three SNPs in the 5′ regulatory region were found (Fig. 5C). A SNP (NW_013185696.1179704 T > C) was found to be different in Chinese and European domestic goose populations. This SNP was not found in the swan geese and Chinese domestic goose populations, but was homozygous in greylag goose and European domestic geese, and was different among individuals in Yili goose population. This SNP was homozygous in YL1, YL4, YL5, YL6, and YL9, heterozygous in YL2, YL7, YL8, and YL10, and not found in YL3. Using JASPAR to predict transcription factor binding sites for the 5′ regulatory region sequence, the results showed that this SNP changed the transcription factor binding sites in the 5′ regulatory region of *IGF-1,* including SRY, ISL2, EGR2, HES family, KLF family, and SP family (Additional file 1: Table S8). The mutation caused the occurrence of a binding site for transcription factor Sp1 (Specificity protein 1) in the 5′ regulatory region of *IGF-1*, and the predicted sequence of the Sp1 binding site was 5′-gcagacacgcacaca-3′ with a relative score higher than 0.8.

## Discussion

In this study, we investigated the domestication history of Chinese and European domestic geese using whole-genome resequencing data from 14 domestic breeds and two wild populations. The results showed that the Chinese domestic geese were domesticated from the swan geese, and the European domestic geese were domesticated from the greylag geese. The Yili goose, which is distributed in Xinjiang, China, originates from the greylag geese. Gene flow was detected between Chinese domestic geese and Yili geese, and the direction of introgression and resulting effects were inferred. This study is the first to reveal the phylogenetic relationship and domestication history of the domestic geese with the swan geese and greylag at the genome-wide level and provides inferences on the causes of body size changes in the Yili geese.

Two clusters were obtained from the phylogenetic tree, the swan geese with the Chinese domestic geese cluster, and the greylag geese with the European domestic geese and the Yili geese cluster. This confirms the hypothesis that domestic geese have two origins. Our results are consistent with those of previous studies using mitochondrial data to determine the origins of Chinese and European domestic geese [20, 54]. In 2020, Heikkinen et al. used genomic data to show that the ancestor of European domestic geese was Greylag goose [25], which is consistent with our results. The topological structure of Yili geese in the genome-wide ML tree is also noteworthy, where Yili geese clustered as a group, but two individuals clustered with Landaise geese, possibly because Yili geese underwent hybridization events with Landaise geese. A similar phenomenon has been observed with the Tibetan chicken, which is a local Chinese chicken breed [55]. The two domestication clusters were also evident in the PCA and ADMIXTURE. These population structure analyses showed that the European domestic goose populations have genetic components of wild geese and Chinese domestic geese, which is maybe due to the often observed interbreeding of geese [25, 54]. Additionally, they showed that Yili individuals contained genetic components from Chinese domestic geese, to varying degrees. This may be an introgression event due to increased geographic proximity causing crossbreeding between Yili geese and Chinese domestic geese.

DaDi was used to estimate the demographic history of Chinese and European domestic geese, and showed that the domestication of Chinese domestic geese occurred approximately 3499 years ago. This is consistent with ancient Chinese records, as there was an official position for breeding livestock and geese during the Zhou Dynasty (1046–256 BC) [13]. Domestication of poultry is generally considered to have occurred earliest in chickens and latest in ducks [56]. Domestic chickens were originated from red jungle fowl subspecies ~ 8000 years ago [56, 57], and ducks in East Asia were domesticated in China before 2200 BP [8]. However, recently published archaeological research has found goose bone fossils in a 7000-year-old cultivation village in the lower Yangtze River, China [14]. Unfortunately, the study did not extract ancient DNA for analysis. The effective population size of swan geese was larger than that of Chinese domestic geese, which is in agreement with the biological phenomenon that the effective population size of wild geese is larger than that of domesticated geese under long-term artificial selection [8]. The effective population size of greylag geese was smaller than that of European domestic geese, which differs from the results of Heikkinen et al. [25]. This may be because of the small number of greylag geese samples in the study, resulting in the biased estimation [58]. In our study, the domestication of European domestic geese occurred approximately 7552 years ago. In 2020, Heikkinen et al. concluded that the split between greylag geese and European domestic geese occurred at 14,000 BCE [25]. Heikkinen et al. noted that their inferred domestication time has large confidence intervals (2014.45–6503.75 generations). The domestication time inferred in our study was 3770.53–3781.13 generations. Therefore, our inferred time range for European geese domestication is consistent with that of Heikkinen et al. [25]. Additionally, the mutation rate and generation interval used were not the same as those used by Heikkinen et al., and the setting of the two parameters is important for explaining the results of the demographic analysis [43, 59]. Meanwhile, using the reference genome of *A. cygnoides* to obtain a variant dataset for European domestic geese may lead to the absence of specific variants in *A. anser.* Assembly genome of the greylag geese and expansion of the sampling range of European domestic geese may improve the accuracy of the estimated divergence time.

We found that gene flow existed between Chinese and European domestic geese and their wild ancestors. This finding supports the widespread occurrence of introgression in birds, especially among goose species [59], and provides a basis for subsequent introgression analyses. Our results showed introgression from Chinese domestic geese to the Yili geese, and 1265 candidate protein-coding genes were obtained, including gene related to neurodevelopment, cell signaling, transcription, translation, and skeletal development.

We identified *IGF-1* located in NW_013185696.1 (119,577—179,398 bp) by annotation of the candidate genes. *IGF-1* is a member of the IGF family, which plays an important role in cell differentiation, proliferation, individual growth, and development and is also the main mediator of growth hormone (GH) to initiate growth activity [60]. The gene structure and expression of *IGF-1* have a direct impact on animal growth and development, and have been reported in humans [61], pigs [62], mice [63], and birds [64].

The basic characteristics of the *IGF-1* gene in chickens are essentially the same as those in mammals, with a length of 50 kb; it is shorter than the human *IGF-1* gene, which is 70 kb in length [65]. Some studies have cloned the mRNA sequence, 5′ regulatory region, and coding region of the *IGF-1* gene in geese and found that the homology of the *IGF-1* gene in geese, chicken, and duck is > 95%, indicating that this gene is highly conserved in poultry [51, 66, 67]. Additionally, Mittanck et al. found that the 5′ untranslated region (5′UTR) upstream of exon 1 of the *IGF-1* gene is of great significance for high-level basic gene transcription [68]. Therefore, research on the polymorphism of the 5′ regulatory region plays an important role in understanding the transcription and expression levels of *IGF-1*. In the current study, we detected three SNP loci in the 5′ regulatory region of the *IGF-1* gene, one of which was located 569 bp upstream of exon 1 present in greylag goose and European domestic geese, but absent in swan geese and Chinese domestic geese, and with different forms in individual Yili geese. The mutation was completely associated with the European domestic goose and also indicated the introgression of Chinese domestic geese into the Yili geese, suggesting that this was possibly a causative mutation located in the 5′ regulatory region of *IGF-1*. The mutation causes the transcription factor binding site to be altered; the binding site for transcription factor Sp1 was present at the mutation site in greylag goose and European domestic geese, while it was absent in swan geese and Chinese domestic geese. Sp1 is an important member of the Sp family, which is a DNA-binding protein containing zinc-finger structures. Sp1 is a general transcription factor that recognizes and binds to GC boxes [69]. Our study indicated that a polymorphic site c.-306 T > C in the 5′ regulatory region of the *IGF-1* gene was associated with the Sp1 binding site. Sp1 plays an important regulatory role in many housekeeping genes [70, 71] and is a *trans*-activator with four domains [72], two of which are glutamine-rich and can act as strong activation domains [73]. Zhu et al. found that Sp1 is involved in the transcriptional regulation of *IGF-1* in rats and that *IGF-1* expression is decreased in cells with a mutation in the Sp1 binding site, indicating the importance of Sp1 in activating *IGF-1* expression [74]. Wang et al. found that *IGF-1* c.-366 A > C is associated with fat deposition in chickens and that this SNP affects *IGF-1* expression through differences in transcription factor binding [75]. Tang et al. found different levels of *IGF-1* expression in goose breeds of different sizes, with significantly higher levels in large geese compared with that in small geese [76]. In summary, we suggest that introgression of Chinese domestic geese into Yili geese affects *IGF-1* expression levels by increasing transcription factor Sp1 binding sites, which influence the body size of Yili geese.

The candidate genes associated with body size were *SOX4, SALL3,* and *SIRT1*. *SOX4* knockdown reduced brain and body size in *Xenopus* embryos [77]. *SALL3* is associated with small body size in Chinese local chicken breeds [78]. Polymorphisms in the *SIRT1* promoter region may modify fat mass and body size in cattle [79]. Therefore, we infer that the change in the body type of Yili geese is due to introgression from Chinese geese. Yili geese body size was similar to that of greylag goose, with an oval shape, short neck, thick and short legs, and adept at flying. Most Chinese domestic geese are smaller than Yili geese, with a boat-shaped body, slender neck, and longer legs than Yili geese. In recent years, there has been a trend that the body type of Yili geese has changed to Chinese domestic geese. Our study suggests that the cause of this phenomenon may be the introgression of several genes related to body size and skeletal development from Chinese domestic geese into Yili geese.

We found that the introgression events were of different proportions in Yili individuals by constructing the ML tree for the *IGF-1* gene sequences located on scaffold NW_013185696.1 and comparing this with the genome-wide tree. This result is consistent with the results of the ADMIXTURE and PCA analyses. This also indicated that the introgression event was incomplete in the Yili population; therefore, and further studies can be performed to elucidate gene introgression within Yili geese.

## Conclusions

In this study, we describe the origins, timing and introgression of domestic geese. Our study was based on deep whole-genome sequencing of 74 individuals from multiple wild and domestic populations. Using this dataset and a suite of cutting-edge population genomic and functional genetic analyses, we showed that the domestic geese have two origins: the ancestor of the Chinese domestic geese is the swan geese, and the ancestor of the Yili geese and European domestic geese is the greylag geese. We also identified the timing of domestication of domestic geese; the domestication of the Chinese geese occurred approximately 3499 years ago, and that of the European geese occurred approximately 7552 years ago. Furthermore, we found that gene flow occurs between domestic geese and their wild ancestors, and between domestic goose species of different origins. Introgression analysis showed that Yili geese had been introgressed by the Chinese domestic geese, and the body size of Yili geese could be influenced by introgression events of some growth-related genes, including *IGF-1*. Therefore, our results have important implications for broader questions on the evolution of complex phenotypes and interspecific introgression.

## Supplementary Information

**Additional file 1: Table S1.** Sample sources of domestic geese; **Table S2.** Summary of genome sequencing and mapping statistics; **Table S3.** Summary of SNPs and indels; **Table S4.** Results of *D* statistics; **Table S5.** Gene names in top 5% introgression regions; **Table S6.** Total GO terms of genes located in top 5% introgression regions; **Table S7.** The parameters estimated in the demographic model; **Table S8.** Prediction of transcription factor binding sites in the *IGF1* 5′ regulatory region.

**Additional file 2: Fig. S1.** Demographic history of domestic geese and their wild ancestors; **Fig. S2.** Site frequency spectrum of swan and Chinese domestic geese populations; **Fig. S3.** Site frequency spectrum of populations of greylag and European domestic geese; **Fig. S4.** Regions of *SIRT1* with introgression between Chinese domestic and Yili geese individuals; **Fig. S5.** Regions of *SALL3* with introgression between Chinese domestic and Yili geese individuals; **Fig. S6.** Regions of *GJA5* with introgression between Chinese domestic and Yili geese individuals.

## Data Availability

Whole-genome resequencing analysis of 18 Chinese domestic geese available from NCBI (BioProject ID: PRJNA662026). The data for 35 other Chinese domestic geese, Landaise geese, Yili geese, and Swan geese are available from NCBI (BioProject IDs: PRJNA767757 and PRJNA817006). The greylag geese data were obtained from a previous study [28]. Roman and Hortobagy geese data are available from NCBI (BioProject ID: PRJNA722049).

## Abbreviations

AIC: Akaike Information Criteria
CDS: Coding sequence
CI: Confidence interval
EPAS1: Endothelial PAS domain protein 1
GJA5: Gap junction protein alpha 5
GO: Gene ontology
IGF-1: Insulin-like growth factor-1
LD: Linkage disequilibrium
ML: Maximum likelihood
N_e_: Effective population size
PCA: Principal component analysis
SALL3: Spalt like transcription factor 3
SIRT1: Sirtuin 1
SOX4: SRY-Box transcription factor 4
Sp1: Specificity protein 1
5'UTR: 5' untranslated region
