# Supplementary material for: Origins, timing and introgression of domestic geese revealed by whole genome data
Source: J Anim Sci Biotechnol. 2023 Feb 13;14:26. doi: 10.1186/s40104-022-00826-9 (PMC9926862; doi:10.1186/s40104-022-00826-9)
Supplement: Supplementary file 2 — Additional file 2: Fig. S1. Demographic history of domestic geese and their wild ancestors; Fig. S2. Site frequency spectrum of swan and Chinese domestic geese populations; Fig. S3. Site frequency spectrum of populations of greylag and European domestic geese; Fig. S4. Regions of SIRT1 with introgression between Chinese domestic and Yili geese individuals; Fig. S5. Regions of SALL3 with introgression between Chinese domestic and Yili geese individuals; Fig. S6. Regions of GJA5 with introgression between Chinese domestic and Yili geese individuals. [file 40104_2022_826_MOESM2_ESM.pdf]

Supplementary Figure

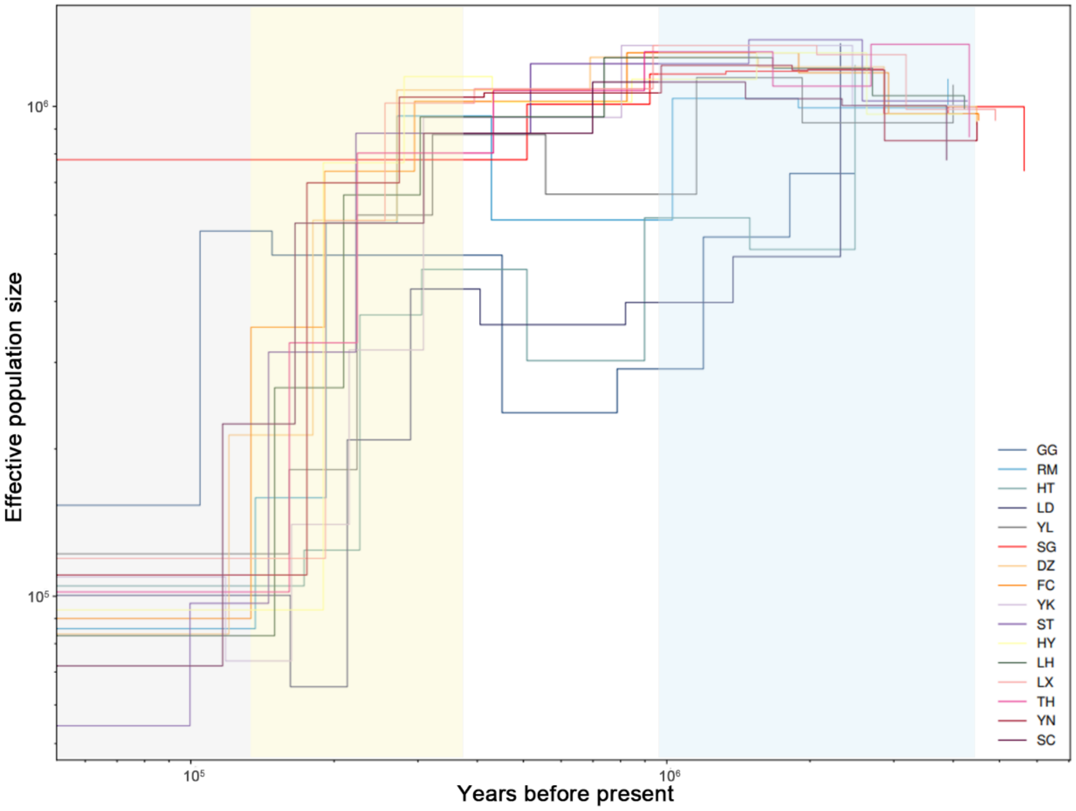

Fig. S1. Demographic history of domestic geese and their wild ancestors. Inferring effective population size using SMC++. Early Pleistocene shown in blue, Mid-Brunhes event shown in yellow, Last Glacial Period (LGP) shown in light grey. The time division was modified from Kozma et al [1].

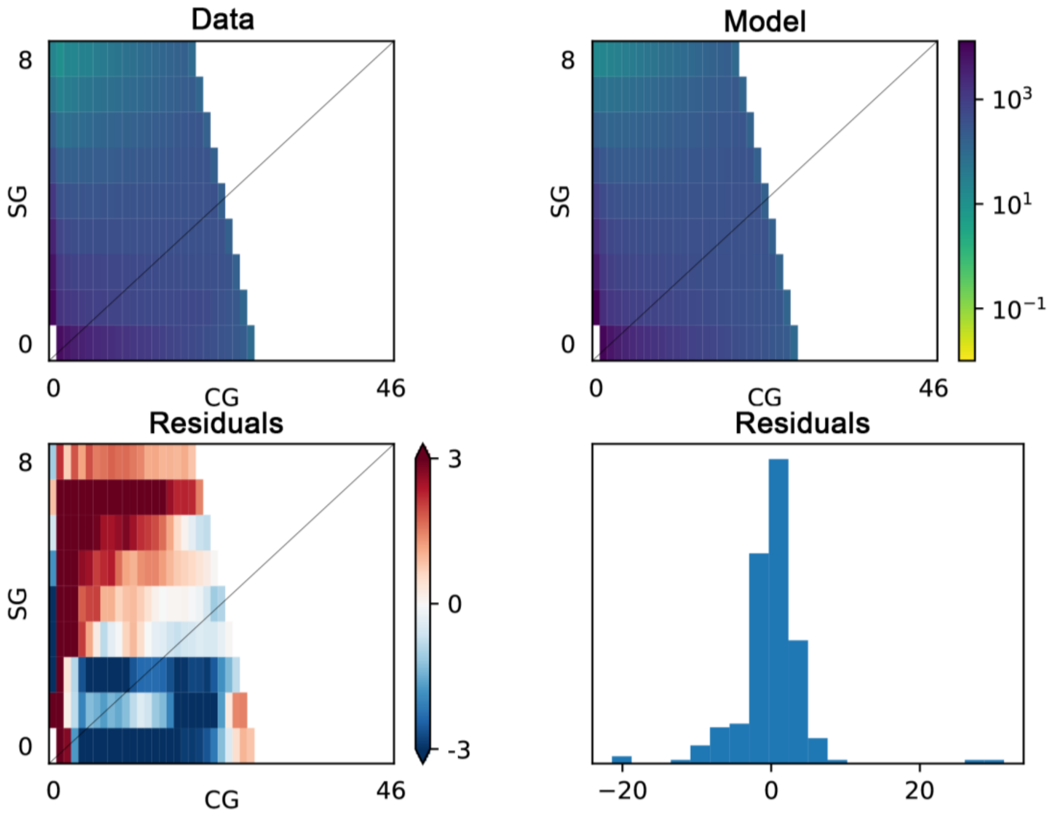

Fig. S2. Site frequency spectrum of the populations of swan and Chinese domestic geese, the first row shows the frequency spectrum of the data and the best model, and the second row shows the residuals between the data and the best model for each bin in the spectrum.

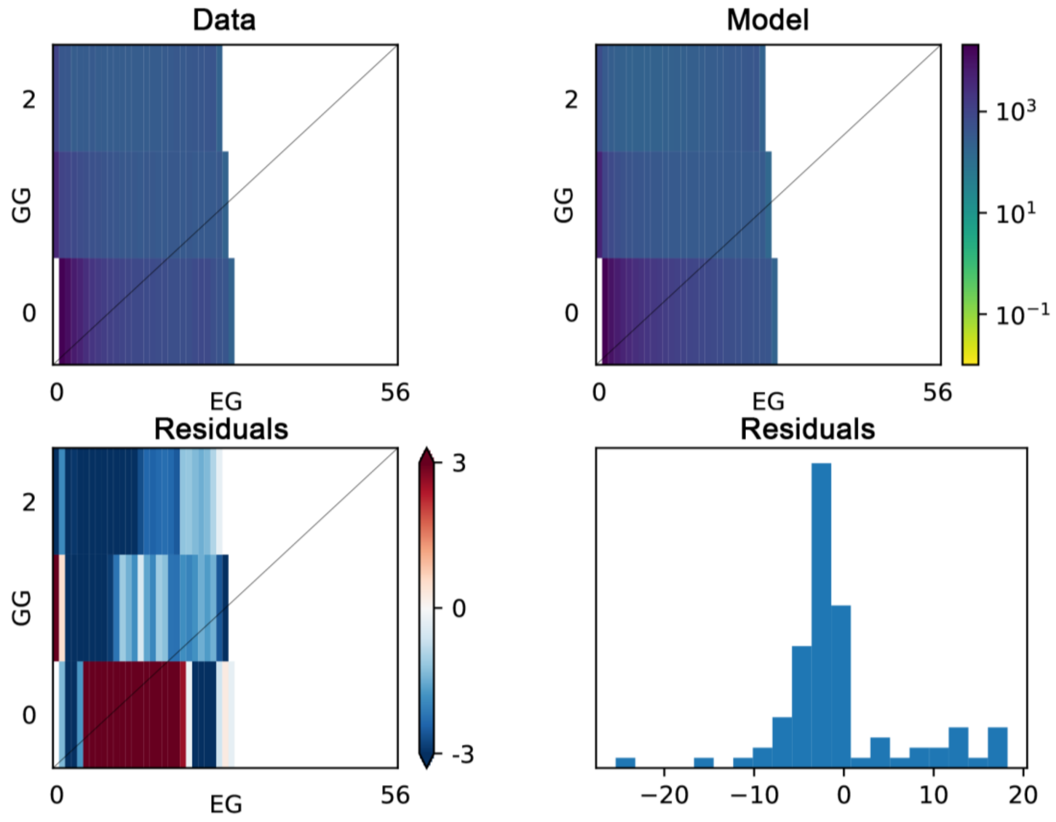

Fig. S3. Site frequency spectrum of the populations of greylag and European domestic geese, the first row shows the frequency spectrum of the data and the best model, and the second row shows the residuals between the data and the best model for each bin in the spectrum.

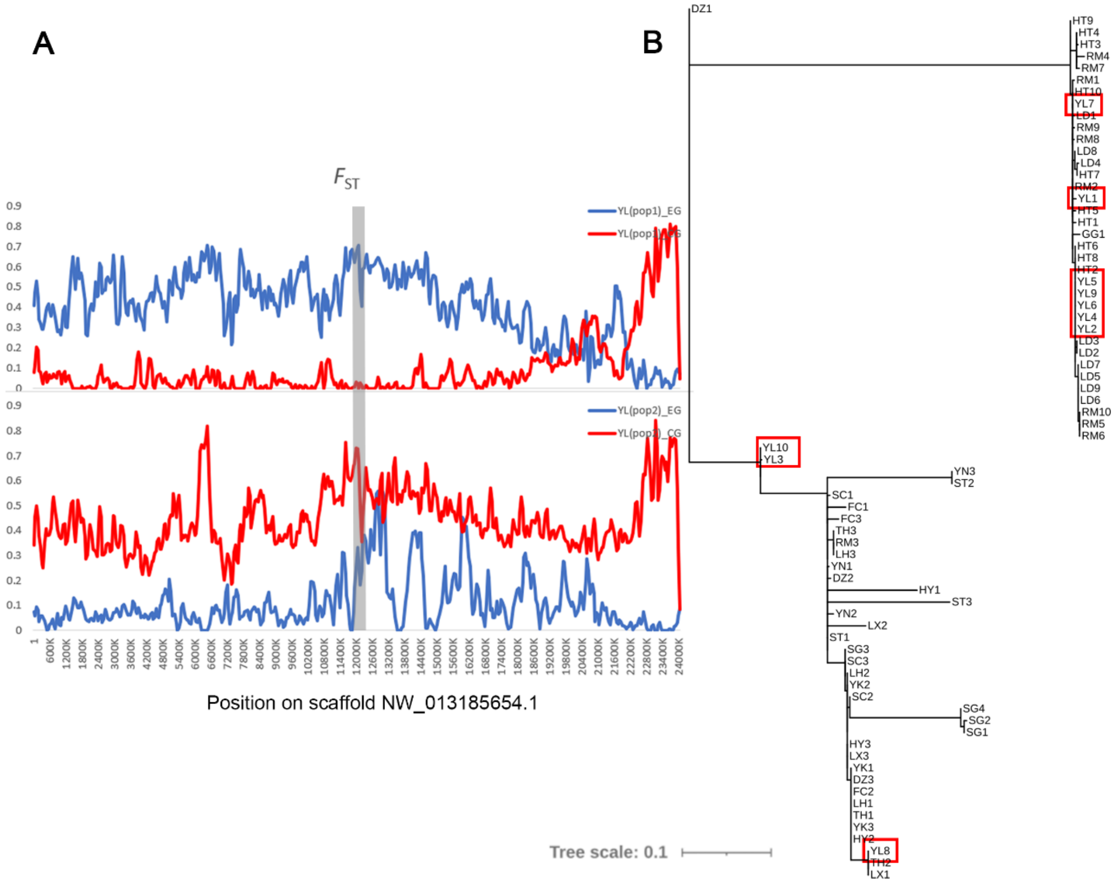

Fig. S4. Regions of *SIRT1* with introgression between Chinese domestic and Yili geese individuals. (A) F_{ST} between Yili goose individuals and the Chinese and European domestic goose populations across scoffold NW_013185654.1. Blue line represents F_{ST} between Yili goose individuals and Landaise geese, and red line represents F_{ST} between Yili goose individuals and Chinese domestic geese. (B) NJ tree constructed by *SIRT1* sequences of region scoffold NW_013185654.1 11939468-11959749. The red frame highlights the branches of Yili goose individuals.

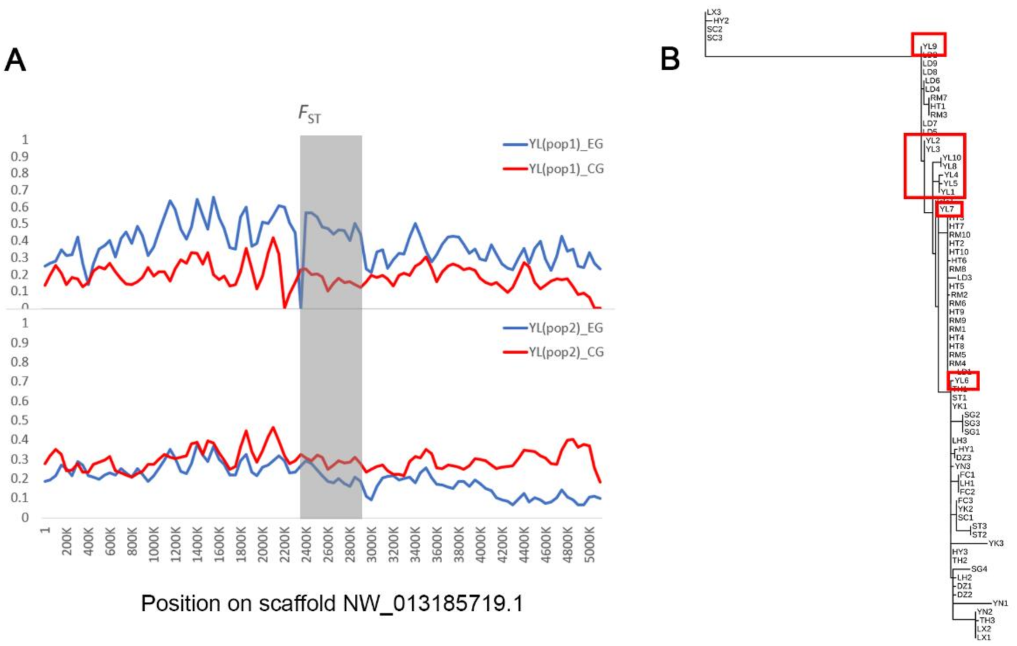

Fig. S5. Regions of *SALL3* with introgression between Chinese domestic and Yili geese individuals. (A) F_{ST} between Yili goose individuals and the Chinese and European domestic goose populations across scoffold NW_013185719.1. Blue line represents F_{ST} between Yili goose individuals and Landaise geese, and red line represents F_{ST} between Yili goose individuals and Chinese domestic geese. (B) NJ tree constructed by *SALL3* sequences of region scoffold NW_013185719.1 2253106-2275099. The red frame highlights the branches of Yili goose individuals.

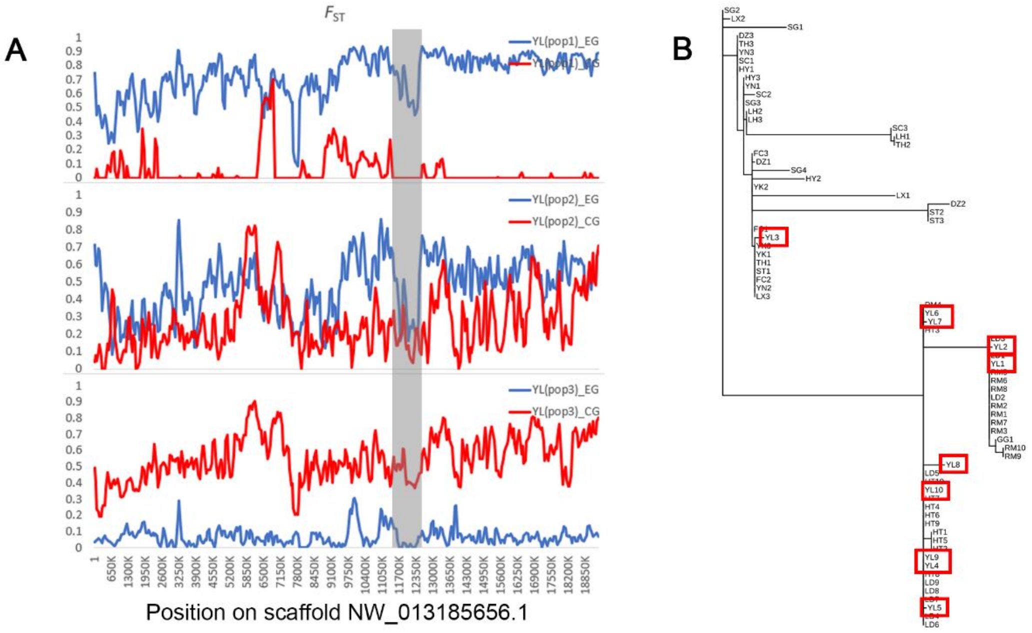

Fig. S6. Regions of *GJA5* with introgression between Chinese domestic and Yili geese individuals. (A) F_{ST} between Yili goose individuals and the Chinese and European domestic goose populations across scoffold NW_013185656.1. Blue line represents F_{ST} between Yili goose individuals and Landaise geese, and red line represents F_{ST} between Yili goose individuals and Chinese domestic geese. (B) NJ tree constructed by *GJA5* sequences of region scoffold NW_013185656.1 12585990-12603816. The red frame highlights the branches of Yili goose individuals.

References

1. Kozma R, Melsted P, Magnusson KP, Hoglund J. Looking into the past - the reaction of three grouse species to climate change over the last million years using whole genome sequences. Mol Ecol. 2016; 25(2):570-80. https://doi.org/10.1111/mec.13496.
